# Risk factors for the development of degenerative changes among patients undergoing rotator cuff repair: A systematic review

**DOI:** 10.1177/17585732211047225

**Published:** 2021-10-18

**Authors:** Matthew Macciacchera, Salwa Siddiqui, Kajeandra Ravichandiran, Moin Khan, Ujash Sheth, Jihad Abouali

**Affiliations:** 1 8863 Royal College of Surgeons in Ireland, Dublin, Ireland; 2 3710 McMaster University, Hamilton, Canada; 3 7938 University of Toronto, Toronto, Canada

**Keywords:** degenerative joint disease, long-term, osteoarthritis, rotator cuff, shoulder surgery, **Level of Evidence** IV

## Abstract

**Background:**

Osteoarthritis (OA) of the glenohumeral joint results in significant pain and functional limitations. It is unclear which risk factors increase the risk of developing glenohumeral OA amongst Rotator Cuff Repair (RCR) patients. The purpose of this systematic review was to examine the risk factors which may contribute to the development of osteoarthritic changes post-operatively.

**Methods:**

MEDLINE, Embase, and PubMed databases were searched to identify studies reporting on demographics of patients who develop OA following RCR.

**Results:**

Seventeen articles were identified investigating a total of 1292 patients. The overall quality of evidence was low. Pooled assessment of OA incidence following RCR at minimum 5 years follow-up found 26% of patients developed OA. Patients requiring revision surgery following retears developed OA at a rate of 29%. Surgical technique and patient demographics may also contribute to degenerative changes.

**Discussion:**

This review found correlations between the aforementioned risk factors and glenohumeral joint degeneration at long-term follow-up after RCR. These findings suggest that future long-term studies should aim to identify prognostic factors that may place a patient at increased risk of developing OA. Such data can be used to counsel patients with respect to long-term outcomes following surgical intervention.

## Introduction

Rotator cuff disease is one of the most common musculoskeletal disorders. It is estimated that over 50% of individuals over the age of 60 have some degree of tearing to the rotator cuff.^1^ Surgery to repair the rotator cuff is increasingly common in North America, particularly arthroscopic minimally invasive interventions. From 1996 to 2006, the number of arthroscopic procedures in the United States increased by 600%.^1^ With a growing number of patients undergoing RCRs, understanding the long-term sequalae following the procedure has become increasingly important. Rotator cuff disease results in a cycle of altered biomechanics and nutrient balance to the glenohumeral joint. It is unclear what role surgery plays with respect to the disease process. Concern exists regarding a potential association with surgical intervention and subsequent development of degenerative changes to the glenohumeral joint. Post-operative degeneration leading to glenohumeral joint osteoarthritis (GHJOA) among patients who have undergone RCR is reported to be as high as 20%.^2^ However, little is known about the risk factors associated with the incidence of GHJOA in these patients. While this association maybe the result of degenerative changes taking place in the cycle of rotator cuff disease it is important to identify factors that may predispose patients towards the development of shoulder arthritis following surgical intervention.

The primary objective of this study was to perform a systematic review of studies with long-term follow-up of RCR patients. The purpose of this review was two-fold: (1) determine the clinical and radiological relationship between RCR and glenohumeral OA, (2) determine what intrinsic or extrinsic risk factors are correlated with the deterioration of the glenohumeral joint.

## Methods

We conducted a systematic review in accordance with the Preferred Reporting Items for Systematic Reviews and Meta-Analyses (PRISMA) guidelines.

### Search strategy

A systematic literature search was performed using the electronic databases Embase, PubMed and MEDLINE from database inception to the fifth week of June, 2020. Medical subject heading (MeSH) terms *rotator cuff repair, shoulder injury, osteoarthritis, long-term* and *pain* were used as keywords and combined using the Boolean operators “AND” and “OR”. (Appendix 1). Two reviewers (MM, SS) independently screened the titles, abstracts and full-text articles resulting from the searches. Any disagreements were addressed by discussion between the 2 reviewers and the senior author (MM, SS and JA) when necessary. The references of the included studies were also reviewed to identify additional articles that were not found by the initial search strategy.

### Eligibility criteria

Studies were eligible for inclusion in this review if they met the following criteria: (1) patients who underwent open or arthroscopic RCR, (2) patients were at least 18 years of age, (3) OA included as a study outcome, and (4) minimum 5-year follow-up. Studies were excluded if they included revision surgeries or concomitant surgeries. All non-English studies and conference abstracts were excluded. Studies were also excluded if data was included from the same study population as another study with longer follow-up.

### Data extraction

Extracted data from eligible studies was summarized using spreadsheet software and a standardized collection form. Information collected included study characteristics (first author, year of publication, level of evidence, study design, surgical technique, tear type), participant demographics (population size, age, sex), length of follow-up, OA rates, risk factor examined, functional and radiographic outcomes.

### Quality assessment

The methodological index for non-randomized studies (MINORS) score was used to assess the quality of studies included in the review.

### Statistical analysis

Descriptive statistics are presented in absolute frequencies with percentages or weighted means with measures of variance where applicable. Agreement between reviewers was evaluated using the Cohen kappa statistic (κ) at all screening stages. Agreement was classified a priori as follows: κ of 0.81–0.99 was considered nearly perfect agreement; κ of 0.61–0.80 was substantial agreement; κ of 0.41–0.60 was moderate agreement; 0.21–0.40 fair agreement and a κ value of 0.20 or less was considered slight agreement.

## Results

### Literature search

**Figure 1.**
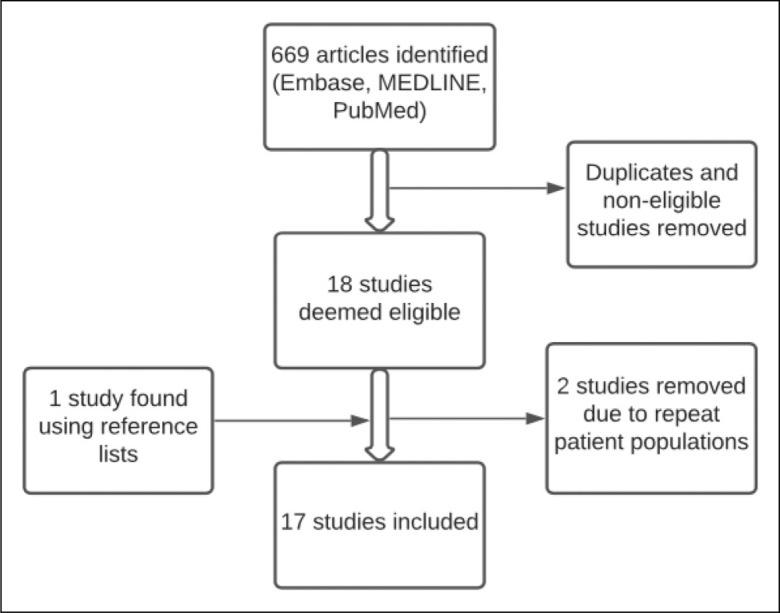
Flow diagram outlining study selection and screening to determine eligibility.

Our literature search yielded 669 studies for review. Following application of inclusion and exclusion criteria 18 studies which reported on the incidence of OA following RCR, with a minimum of five years follow-up underwent full text review. Two studies were excluded^3,4^ given subsequent evaluation on identical patient populations.^5^ Additionally, one article was found using the reference lists of included studies^6^ which resulted in a total of 17 articles included in this systematic review (Figure 1).

### Study characteristics and patient information

Ten of the 17 of the studies included in this review were conducted with a retrospective study design. The total number of patients from these studies was 1292. Studies were published over the past 15 years. 14 studies were published in North America and the remainder were published in the United Kingdom and Netherlands (2).

Clinical outcomes for 1370 shoulders were included along with radiological findings for 1298 shoulders (Table 1). Twelve studies included open RCR patients (684 shoulders), three studies included arthroscopic RCR patients (139 shoulders) and two considered patients who underwent either procedure (547 shoulders). Twelve studies reported patient sex: 902 patients, 506 males and 396 females (56% and 44%, respectively).

**Table 1.** Study characteristics. For shoulders studied, clinical population size listed first and radiological population size listed in brackets.

*Authors*	*Year Published*	*Study Design*	*Level of Evidence*	*Shoulders Studied*
**Bartl et al.** ^7^	2012	Retrospective	4	25
**Collin et al.** ^8^	2019	Retrospective	4	66 (45)
**Collin et al.** ^9^	2020	Retrospective	4	53 (36)
**Elia et al.** ^10^	2017	Cohort	4	53
**Flurin et al.** ^5^	2017	Retrospective	4	401
**Herve et al.** ^11^	2019	Retrospective	4	126
**Jeong et al.** ^6^	2019	Retrospective	3	146
**Jost et al.** ^12^	2006	Retrospective	4	20
**Matsuba et al.** *^13^*	2018	Retrospective	4	86
**Matsuba et al.** ^14^	2019	Retrospective	4	68
**Nich et al.** ^15^	2009	Therapeutic	4	44
**Paxton et al.** ^16^	2013	Therapeutic	4	15
**Plachel et al.** ^17^	2019	Retrospective	4	20
**Plachel et al.** ^18^	2020	Cohort	3	22
**Randelli et al.** ^19^	2019	Cohort	3	102 (101)
**Vastamäki et al.** ^20^	2013	Therapeutic	4	100 (67)
**Zumstein et al.** ^21^	2008	Cohort	4	23

### Risk factors for OA progression

#### Age

Four studies reviewed attempted to correlate the age of their patients with the likelihood of developing OA post-operatively. Matsuba et al.^13^ did not find that older patients were more likely to show OA progression (*p* = 0.26). Herve et al.^11^ reported similar non-significant findings (*p* = 0.1), as did Plachel et al.^17^ (*p* = 0.547). However, Flurin et al.^5^ found that mean age at surgery was significantly higher in their post-operative osteoarthritic group (*p* < 0.0001).

#### Sex

Three studies reported on the relationship between patient sex and OA progression. Matsuba et al.^13^ found the relationship to be non-significant (*p* = 0.26), as did Herve et al.^11^ (*p* = 0.6). Flurin et al.^5^ once again found there was a significant relationship to OA progression, with a higher proportion of males developing the disease than females (76% vs 57%, *p* = 0.004).

#### Tear type

Herve et al.^11^ and Flurin et al.^5^ found the initial size and type of tear experienced by patients to be significant prognostic factors for OA development. Patients studied by Herve et al.^11^ were significantly less likely to develop OA if their supraspinatus (SSN) tendon integrity was maintained (*p* < 0.0001). Massive tear of three tendons was more frequently seen in their patients who did develop OA (*p* = 0.04). Flurin et al.^5^ determined that a greater degree of fatty infiltration was correlated with OA following tears of the infraspinatus (ISN, *p* = 0.0006), subscapularis (SSC, *p* < 0.0001) and SSN (*p* = 0.01) tendons. Muscle atrophy was also significantly higher in OA patients with SSN and ISN tears. Superior humeral head migration and superior glenoid wear are the main pathological changes which define the pattern of OA observed in these studies.

#### Surgical technique

Twelve of the eligible studies in this review included data specifically for patients who underwent open RCR. Elia et al.^10^ reported increased osteoarthritic lesions in 69% of their cases at last follow-up, and a Samilson-Prieto grade of 0 in only 21% of their cases (*p* = 0.041). Flurin et al.^5^ compared outcomes for patients who underwent open or arthroscopic RCR. The proportion of patients with OA was 26% for those treated by open surgery and 14% for those treated by arthroscopic surgery, at last follow-up (*p* = 0.02). However, tears involving three tendons accounted for 10% of the open procedures but only 3% of the arthroscopic procedures (*p* = 0.0001).

#### Retear and loss of integrity

Failure of the surgically repaired tendon(s) was significantly correlated with increased OA progression in four studies. The incidence of retears among RCR patients in studies which reported retear rates was 29%. Flurin et al.^5^ found that the presence of OA was higher amongst patients with unhealed or retorn cuffs than those with fully healed cuffs (46% vs. 25%; *P* = 0.012). Patients studied by Plachel et al.^18^ were significantly more likely to show OA progression when tendon integrity failed post-operatively (*p* = 0.040). Bartl et al.^7^ found that the cross-sectional areas of rotator cuff muscles were greater in patients when repair was intact (*p* < 0.05). Paxton et al.^16^ studied clinical and radiological outcomes in patients with repair failure. These authors found that SSN retear (*p* = 0.0001) and massive tears of the rotator cuff (*p* = 0.04) were both significant risk factors for the development of glenohumeral OA.

#### Length of follow-up

In two of the studies which compared rates of OA at initial follow-up and last follow-up, the incidence in patients was higher at the later date. Matsuba et al.^13^ determined OA was significantly worse at 10 years than 1 year post-operatively (*p* = 0.001). Bartl et al.^7^ also determined the average degree of presenting OA increased significantly at last follow-up (*p* < 0.05). Studies which did not find the progression of OA to be significant may not have followed patients for long enough, or had too few patients. For example, Jost et al.^12^ determined there was no progression in the stage of glenohumeral OA between 3.2 and 7.6 years (*p* = 0.062) among the 20 patients in their study.

### Radiological findings

The pooled mean incidence of GHJOA among eligible studies was 26%. The study authors included MRI results for their patients according to the Samilson-Prieto classification system,^22^ with the exception of Vastamäki et al.^20^ The Samilson-Prieto system calls for grading of glenohumeral arthrosis pre and post-operatively, to allow for comparison.^22^ Shoulder OA is classified as normal, mild, moderate, or severe, to allow for assessment of progression in each patient. Narrowing of the glenohumeral space is pathognomonic for the progression of degenerative change in the glenohumeral joint.

Matsuba et al.^14^ used MRI to observe rotator cuff integrity and how it related to progression through the Samilson-Prieto classification system. Incidence of OA in the affected shoulder was significantly higher than that of the unaffected shoulder (*p* < 0.001). These authors also found that cuff integrity was significantly correlated with disease progression (*p* = 0.0024), for the affected side: a greater percentage of the patients with “poor” cuff integrity developed OA, compared to those with “good” cuff integrity. Flurin et al.^5^ found that a lower post-operative strength score was more likely to be found in patients with higher Samilson-Prieto grades (*p* = 0.008). Jeong et al.^6^ determined the progression of OA by classifying patients into two groups: 1) pre-operative mild OA and 2) non-OA groups. In the mild OA group, 26.1% of patients who experienced retears went on to develop severe OA, compared to only 5.9% among healed cases. The odds ratio comparing these variables was high (5.65) and significant in the OA group (*p* = 0.022), but not in the non-OA group (*p* = 0.703). Finally, Plachel et al.^17^ found that secondary OA progressed significantly from the pre- to post-operative period (*p*​ = 0.003) (Table 2).

**Table 2.** Risk factor rates and radiological data. Mean values used for length of follow-up and patient age. Patient age considered at time of surgery. Incidence of retears and the development of GHJOA measured at last follow-up.

*Authors*	*Shoulders Imaged*	*Length of Follow-up*	*Patient Age*	*Surgical Technique*	*Tear Size*	*Re-tear rate*	*Incidence of GHJOA*
**Bartl et al.** ^7^	25	5.8 years	NA	Open	Massive	44%	NA
**Collin et al.** ^8^	45	20 years	53 ± 7.8 years (33-73)	Open	Large-to-Massive	47%	6%
**Collin et al.** ^9^	36	20 years	52 years (25-65)	Open	Massive	NA	9%
**Elia et al.** ^10^	53	11.4 years	59 ± 8 years (38-75)	Open	NA	42%	69%
**Flurin et al.** ^5^	401	11 ± 7.8 years	56.3 ± 7.6 years	Both	NA	23%	19%
**Herve et al.** ^11^	126	20 years	52.3 years (25.3–68.6)	Open	Massive	43.8%	29%
**Jeong et al.** ^6^	146	5.5 years	NA	Both	Large-to-Massive	31.1% (OA group)	13%
**Jost et al.** ^12^	20	7.6 years	*66 years	Open	Full thickness	40%	14%
**Matsuba et al.** ^13^	86	11 ± 1 years	60.4 ± 7.3 years	Open	Small-to-Large	5.9%	44%
**Matsuba et al.** ^14^	68	11.4 ± 1.2 years	60.2 ± 9.1 years	Open	Small-to-Medium	NA	55%
**Nich et al.** ^15^	44	7.2 years	59 years	Open	NA	12%	75%
**Paxton et al.** ^16^	15	10 years	*74.6 years (63-90)	Arthroscopic	Massive	57%	80%
**Plachel et al.** ^17^	20	14 ± 3 years	55 ± 8 years (31-68)	Open	Large	29%	25%
**Plachel et al.** ^18^	22	12 ± 1 year	55 ± 8 years (31-68)	Arthroscopic	Full thickness	45%	>50%
**Randelli et al.** ^19^	101	^**^10.64 years	60 years	Arthroscopic	Small-to-Large	34%	14%
**Vastamäki et al.** ^20^	67	20 years	52 years	Open	NA	94%	27%
**Zumstein et al.** ^21^	23	9.9 years	54 years (42-67)	Open	Massive	57%	61%

*Patient age considered at time of last follow-up. ^**^Median value used.

### Clinical findings

All of the studies included in the current review observed clinical improvements in patients who underwent successful RCR. However, only five of these studies evaluated how clinical parameters related to the development of OA amongst their patients (Table 3).

**Table 3.** Clinical findings.

*Authors*	*Mean CMS* *(pre-operatively)*	*Mean CMS* *(post-operatively)*	*Mean Subjective Shoulder Score* *(post-operatively)*
**Bartl et al.** ^7^	42.3	73.1	NA
**Collin et al.** ^8^	44 ± 15.3	68 ± 17.7	73 ± 23
**Collin et al.** ^9^	51.5 ± 14.1	71	77.2 ± 22
**Elia et al.** ^10^	NA	74.7	82.5 ± 20
**Flurin et al.** ^5^	53 ± 14.5	78 ± 13.5	83 ± 17
**Herve et al.** ^11^	45.3 ± 19.6	67.4 ± 18.7	73.5 ± 21
**Jeong et al.** ^6^	NA	^**^87.4 ± 8.2, 89.2 ± 7.8	NA
**Jost et al.** ^12^	NA	88	74
**Matsuba et al.** ^13^	NA	^*^32.9	NA
**Matsuba et al.** ^14^	NA	^*^32.3 ± 3.3	NA
**Nich et al.** ^15^	54.6	74.1	NA
**Paxton et al.** ^16^	NA	65.3	9.2 (6-12)
**Plachel et al.** ^17^	NA	78 ± 10	83 ± 12
**Plachel et al.** ^18^	NA	83 ± 19	84 ± 19
**Randelli et al.** ^19^	NA	78.05	^***^10.44 ± 3.45
**Vastamäki et al.** ^20^	NA	71	^***^7.6
**Zumstein et al.** ^21^	NA	81	82

* UCLA shoulder score indicated.

^**^ CMS shown for OA patients and non-OA patients, respectively.

^***^ Simple Shoulder Test results shown.

The Constant-Murley Score (CMS) is the most common standardized method used for measuring a patient's shoulder function.^23^ Higher scores signify less pain, a greater range of motion (ROM), more strength and the ability to perform normal daily activities. At 20 years follow-up, Herve et al.^11^ determined patients who developed OA were more likely to have an inferior CMS, compared to patients with no arthritic changes (61/100 vs 71/100; *p* = 0.02). Flurin et al.^5^ also found that mean CMS were lower in patients with OA compared to those who did not develop OA, at minimum 10 years follow-up (73/100 vs. 79/100; *p* < 0.001). Additionally, Flurin et al.^5^ reported increased pain (*p* = 0.001) and decreased ROM (*p* = 0.005) were significantly correlated with higher rates of OA.

Vastamäki et al.^20^ found that glenohumeral arthrosis detected by MRI did not correlate with an inferior CMS (*p* = 0.06). Matsuba et al.^14^ also reported that post-operative UCLA shoulder scores showed no significant difference among patients with and without OA progression (*p* = 0.70).

## Discussion

Establishing a relationship between risk factors and the development of OA in RCR patients is a developing field of research. Although the goal of RCR is to restore functionality to the shoulder joint and relieve pain, rates of OA progression post-operatively are consistently reported as higher than those of the general population.^1^ While surgical methods have improved and functional outcomes are now consistently high,^1^ GHJOA remains an outcome of concern.^2^ This study sought to explore the correlations between risk factors and the degeneration of the glenohumeral joint in RCR patients, along with clinical parameters which may be potentially prognostic for degenerative changes in these patients. Risk factors considered consisted of age, sex, tear type, surgical technique, retear and length of follow-up.

Each study included in this review explored and reported on degenerative changes in RCR patients post-operatively, examining varying risk factors of interest However, no review has been published to-date which compares risk factors across studies with consolidated data for a large patient population.

Clinical parameters were identified in the “Results” section which may be useful for the identification of patients at increased risk of GHJOA post-operatively. A large focus in the field of RCR has been on the determinants of clinical outcomes following surgery. Studies included in this review have expanded the scope of their findings to include how such determinants and clinical outcomes correlate with OA progression.^5,11^ The Constant-Murley Shoulder score is the main clinical test which we found to be correlated with OA.^18^ Patients without a significant improvement in their scores in the post-operative period were significantly more likely to demonstrate signs of OA on MRI at last final follow-up. Therefore, the findings from the current study support screening by MRI for the detection of early-onset degeneration in all RCR patients with a low CMS score. Results from this review^4,5,14^ also suggest a significant relationship may exist between patients reporting poor clinical outcomes and OA progression. These findings warrant further exploration to determine the exact nature of the relationship.

Tendon retear and failure of repair were the most commonly reported risk factors for the development of OA among the studies considered. 15 studies reported the number of patients who experienced retears of their surgically repaired cuff, combining for an incidence of 29%. According to Flurin et al.^5^ and Plachel et al.^18^ the presence of osteoarthritic changes were significantly more likely to be found in patients with retorn cuffs and decreased tendon integrity. The cause of retears may be multifactorial: the pattern of rotator cuff tear, method of repair, extent and progression of rehabilitation may influence a patient's degree of risk. Significant use of the repaired joint too early in the recovery process may place patients at significant risk of repair failure, as tendon to bone healing is unlikely to occur before 12 weeks post-operatively.^24^

Glenohumeral joint degeneration is a well-known outcome following traumatic shoulder injury or chronic instability.^21^ Large rotator cuff tears leave the humeral head more prone to subluxation and impaired functional stability. This pathological process explains why higher levels of glenohumeral joint degeneration were seen in patients with larger tears and more tendon involvement.^5,14^ Studies which specifically explored risk factors for OA primarily correlated the size of tears with disease progression.^5,6,13,14,18^ While most studies revealed a positive correlation between tear size and the degree of OA developed, Herve et al.,^11^ Flurin et al.^5^ and Paxton et al.^16^ were able to determine the relationship was significant amongst their patients. Massive tears were more frequently seen in patients who developed OA than those who did not, while increased fatty infiltration and muscle atrophy of the shoulder joint both lead to worse clinical and radiological outcomes. Randelli et al.^19^ found that especially for large symptomatic tears, RCR slowed the progression of OA. Another retrospective analysis of RCR over 10 years reported similar results.^13^ These findings in combination with those mentioned throughout this review support our suggestion that a patient's likelihood of developing OA may be determined through proper clinical and radiological assessment.

## Limitations

This study has several limitations. Due to the retrospective nature of the studies included in the review, the overall quality of evidence was low (level III or IV). As a result, inherent biases associated with retrospective study designs may have been incorporated. Additionally, there was significant heterogeneity among the included studies, which limited our ability to provide an overall pooled estimate. However, the main limitation of this study is the lack of a control group including patients with rotator cuff tears that have not been surgically repaired. Such a patient group would allow for a more accurate estimate of the extent to which RCR and the development of GHJOA are related.

Many of the studies included in this review consider the arthropathy type of OA. Therefore, Samilson-Prieto classification may not be the most appropriate criteria for assessment of disease severity. This is because osteophytosis tends not to be very prominent. Hamada classification may have been more appropriate for this review.

The greatest limitation within the available literature is that there is significant variation in the surgical techniques used for the repair of rotator cuff tears among surgeons, which makes comparison or interpretation of results difficult. The development of OA with increasing length of follow-up may be confounded by expected degenerative changes associated with aging. Finally, a minimum of five-years follow-up may not be long enough to observe osteoarthritic changes especially if RCR was performed at a very young age

## Conclusion

This systematic review aimed to identify the risk factors associated with glenohumeral joint osteoarthritis progression following RCR. Results of this study suggest that there may be a correlation between the occurrence of retears and tendon integrity with the progression of the disease. Further prospective research is needed to establish a causative relationship and better understanding of the associated pathogenesis. Future studies should ensure risk factors are monitored in RCR patients throughout the extended follow-up period. This will allow for more accurate methods of identifying patients with an increased likelihood of developing OA and risk of disease progression.

## Appendix 1: Search strategy

**Table:**

Number	Search Statement	Results
1.	*rotator cuff injury/	3969
2.	*osteoarthritis/	264,408
3.	rotator cuff repair.mp	2257
4.	shoulder joints.mp.	571
5.	long term.mp.	704,792
6.	*shoulder/	7486
7.	shoulder joint*.mp.	20,739
8.	Shoulder Pain/	4826
9.	2 or 4 or 6 or 7 or 8	55,829
10.	1 or 3	5020
11.	5 and 9 and 10	102
